# Genomic insights into the seawater adaptation in Cyprinidae

**DOI:** 10.1186/s12915-024-01885-2

**Published:** 2024-04-19

**Authors:** Ying Wang, Xuejing Zhang, Jing Wang, Cheng Wang, Fei Xiong, Yuting Qian, Minghui Meng, Min Zhou, Wenjun Chen, Zufa Ding, Dan Yu, Yang Liu, Yumei Chang, Shunping He, Liandong Yang

**Affiliations:** 1 Hubei Engineering Research Center for Protection and Utilization of Special Biological Resources in the Hanjiang River Basin, College of Life Sciences, Jianghan University, Wuhan, 430056 China; 2 State Key Laboratory of Freshwater Ecology and Biotechnology, Institute of Hydrobiology, Chinese Academy of Sciences, Wuhan, 430072 China; 3 Academy of Plateau Science and Sustainability, Qinghai Normal University, Xining, 810016 China; 4 University of Chinese Academy of Sciences, Beijing, 100049 China; 5 National and Local Joint Engineering Laboratory for Freshwater Fish Breeding, Heilongjiang Province’s Key Laboratory of Fish Stress Resistance Breeding and Germplasm Characteristics On Special Habitats, Heilongjiang River Fisheries Research Institute, Chinese Academy of Fishery Sciences, Harbin, 150070 Heilongjiang China; 6 School of Biological Sciences, University of Bristol, Life Sciences Building, 24 Tyndall Avenue, Bristol, BS81TQ UK

**Keywords:** Far Eastern dace, Migratory, Osmoregulation, Seawater adaptation

## Abstract

**Background:**

Cyprinidae, the largest fish family, encompasses approximately 367 genera and 3006 species. While they exhibit remarkable adaptability to diverse aquatic environments, it is exceptionally rare to find them in seawater, with the Far Eastern daces being of few exceptions. Therefore, the Far Eastern daces serve as a valuable model for studying the genetic mechanisms underlying seawater adaptation in Cyprinidae.

**Results:**

Here, we sequenced the chromosome-level genomes of two Far Eastern daces (*Pseudaspius brandtii* and *P. hakonensis*), the two known cyprinid fishes found in seawater, and performed comparative genomic analyses to investigate their genetic mechanism of seawater adaptation. Demographic history reconstruction of the two species reveals that their population dynamics are correlated with the glacial-interglacial cycles and sea level changes. Genomic analyses identified *Pseudaspius*-specific genetic innovations related to seawater adaptation, including positively selected genes, rapidly evolving genes, and conserved non-coding elements (CNEs). Functional assays of *Pseudaspius-*specific variants of the prolactin (*prl)* gene showed enhanced cell adaptation to greater osmolarity. Functional assays of *Pseudaspius* specific CNEs near *atg7* and *usp45* genes suggest that they exhibit higher promoter activity and significantly induced at high osmolarity.

**Conclusions:**

Our results reveal the genome-wide evidence for the evolutionary adaptation of cyprinid fishes to seawater, offering valuable insights into the molecular mechanisms supporting the survival of migratory fish in marine environments. These findings are significant as they contribute to our understanding of how cyprinid fishes navigate and thrive in diverse aquatic habitats, providing useful implications for the conservation and management of marine ecosystems.

**Supplementary Information:**

The online version contains supplementary material available at 10.1186/s12915-024-01885-2.

## Background

Fish migration is the movement from one habitat or region to another typically for spawning [1, 2]. There are three styles among migratory fishes: oceanodromous fishes migrating exclusively within seawater, potamodromous fishes migrating exclusively within freshwater, and diadromous fishes migrating between seawater and freshwater [3, 4]. During migration, fish are confronted multiple challenges posed by environmental conditions including temperature, water flow, and salinity [5, 6]. Three hundred sixty-eight species of diadromous fishes are known (http://www.fishbase.cn), including salmonoids, osmeroids, galaxioids, lampreys, sturgeons, and sicydiine gobies [7–9]. However, the Far Eastern daces (*Pseudaspius* species) are among the only diadromous fishes in Cyprinidae, with limited migratory behavior observed in some other cyprinid species, including idle (*Leuciscus idus*), roach (*Rutilus rutilus*), and bream (*Vimba vimba*) [10–12].

Meanwhile, environmental salinity fluctuations exert significant influences on various aspects of fish biology such as osmotic regulation, hormone control, energy metabolism, and growth [13–15]. The Far Eastern daces, *Pseudaspius* species (Pisces, Cyprinidae), speciated and dispersed around the Sea of Japan [16]. The genus *Pseudaspius* encompasses two freshwater resident species (*P. nakamurai* and *P. sachalinensis*) and two diadromous species (*P. hakonensis* and *P. brandtii*), belonging to the subfamily Leuciscinae within the large family Cyprinidae [17]. As migratory species of cyprinid fishes, *P. hakonensis* and *P. brandtii* comprise a unique group with the freshwater-seawater transition [18], which has attracted much research interest. *P. hakonensis* and *P. brandtii* are commonly known as gold beachhead fish and black beachhead fish, respectively, and return from the Japan sea for spawning in Suifen River (Heilongjiang Province in China) from April to July each year [19]. Specifically, they migrate upstream into the estuary in batches after their gonadal maturation, and the parent fish immediately return to the offshore area after spawning; the juveniles grow in the river then they overwinter in the river depths before migrating to the sea [20, 21]. A previous study reported that *P. brandtii* evolved unique strategies and exhibited higher tolerance and adaptability to salinity and alkalinity than other cyprinids [22, 23]. Therefore, the *P. hakonensis* and *P. brandtii* genomes may represent a genetic resource for studies on seawater adaptation in migratory fishes.

Multiple studies have documented the genetic basis of seawater adaptation of salmonid fishes and disentangled osmoregulatory genes and functional groups [24–26]. Recently, genomic studies in salmon and trout strikingly revealed a small genomic region (including *rock1* and *greb1l* genes) was strongly associated with migration behavior and timing [26–29]. Furthermore, simulated experiments conducted on fishes subjected to salinity treatment have revealed insights into the significance of the osmoregulatory gene family, associated metabolic pathways, and gene expression patterns in determining crucial adaptations to seawater [24, 30–32]. For example, the euryhaline Javafish medaka (*Oryzias javanicus*) genome sequencing provided insights into the molecular basis of osmotic regulation and hatching enzyme activity [32]. Nevertheless, the genetic mechanism of *Pseudaspius* species (*P. brandtii* and *P. hakonensis*) adapting to seawater is unclear.

In this study, we focused on two species of cyprinid fishes in seawater, *P. hakonensis* and *P. brandtii*, to unravel the genetic basis of their adaptation to seawater during the migratory process. To achieve this, we first generated high-quality, chromosome-level assemblies of the genomes of *P. hakonensis* and *P. brandtii*. By comparing these genomes with those of other teleosts, we identified genes that underwent positive selection and rapid evolution as well as several conserved non-coding elements (CNEs) that are likely responsible for their seawater adaptation. Importantly, we validated the *Pseudaspius*-specific genetic innovations using functional assays. The findings of this study shed light on the intricate genetic mechanisms underlying seawater adaptation in *Pseudaspius* species. These discoveries serve as a foundation for further investigations into the adaptation of migratory fishes and contribute to our understanding of their remarkable ability to thrive in osmotically contrasting.

## Results

### Genome assembly and genome annotation

**Fig. 1:**
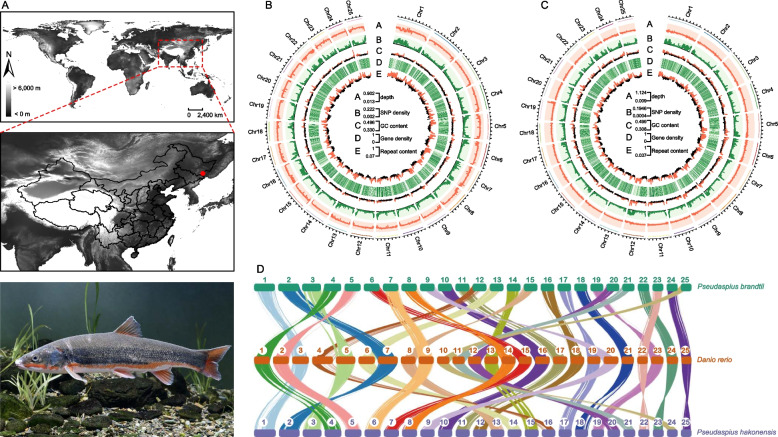
Sampling sites and genome assemblies for *P. hakonensis* and *P. brandtii*. **A** A sampling site (red circle) in Suifen River (Helongjiang province, China) and a photo of the *P. brandtii*. **B** Circos plots showed the distributions of genomic components in *P. hakonensis*; the outer circle was the chromosome. **C** Circos plots showed the distributions of genomic components in *P. brandtii*; the outer circle represents the chromosome. **D** Chromosomal syntenic relationship of *P. brandtii*, *P. hakonensis*, and *D. rerio*. The number (1–25) represented the number of the chromosome

Using Illumina, Nanopore, and Hi-C sequencing, we acquired a total of 236.91 Gb data for *P. hakonensis* and 231.44 Gb data for *P. brandtii*, respectively (Fig. 1A, B, and C, Additional file 1: Table S1). Subsequently, we generated chromosome-level genome assemblies for *P. hakonensis* and *P. brandtii*. The assemblies of *P. hakonensis* and *P. brandtii* genomes were 840.77 Mb and 848.49 Mb, with contig N50 length of 3.68 Mb and 3.63 Mb, GC content of 39% and 38%, respectively (Additional file 1: Table S2 and S3). To further refine the assemblies, we anchored the contigs onto 25 chromosomes with mounting rates of 96.54% for *P. hakonensis* and 97.20% for *P. brandtii*. The genome-wide Hi-C heatmaps showing chromosome crosstalk adhered to the interaction rule, with noticeably stronger signal strength around the diagonal compared to other positions (Additional file 2: Figure S1, Additional file 1: Table S4. This suggests the high quality and completeness of the genome assembly. The summary of genome characteristics of *P. hakonensis* and *P. brandtii* are shown in Fig. 1B, C. Evaluation of genome completeness based on BUSCO identified that 94.50% of the complete BUSCO in the *P. hakonensis* genome assembly including 88.00% of the complete and single-copy genes and 6.50% of the complete and duplicated genes, while 94.90% of the complete BUSCO in the *P. brandtii* genome assembly including 87.90% of the complete and single-copy genes and 7.00% of the complete and duplicated genes (Additional file 1: Table S5). These results confirm the high quality of our genome assemblies for both species, providing a solid foundation for our investigations into the genetic basis of seawater adaptation in *P. hakonensis* and *P. brandtii*.

We conducted an analysis of repetitive sequences and protein-coding genes in the genomes of *P. hakonensis* and *P. brandtii*. Approximately 36.93% of the bases in the *P. hakonensis* genome and 37.20% of the bases in the *P. brandtii* genome were classified as repetitive sequences (Additional file 1: Table S6). We also predicted a total of 23,839 and 24,012 protein-coding genes in *P. hakonensis* and *P. brandtii* genomes, respectively (Additional file 1: Table S7). Approximately 97.54% of *P. hakonensis* and 97.69% of *P. brandtii* predicted genes were successfully annotated using the Non-Redundant Protein Sequence Database (NR), InterProScan, Uniport (Universal Protein), Pfam, and eggNOG (evolutionary genealogy of genes: Non-supervised Orthologous Groups) databases (Additional file 1: Table S8). To gain further insights into the genomic relationships, we conducted a genome collinearity analysis between *P. hakonensis*, *P. brandtii*, and *Danio rerio* [33]. The results showed that these species exhibited good collinearity without any evidence of chromosomal fission or fusion events (Fig. 1D). This corroborates the high-quality and chromosome-level nature of our assembled genomes for *P. hakonensis* and *P. brandtii*, which provide a solid foundation for our investigations into the genetic basis of seawater adaptation in these two Far Eastern daces.

### Phylogenetic relationships and population history

**Fig. 2:**
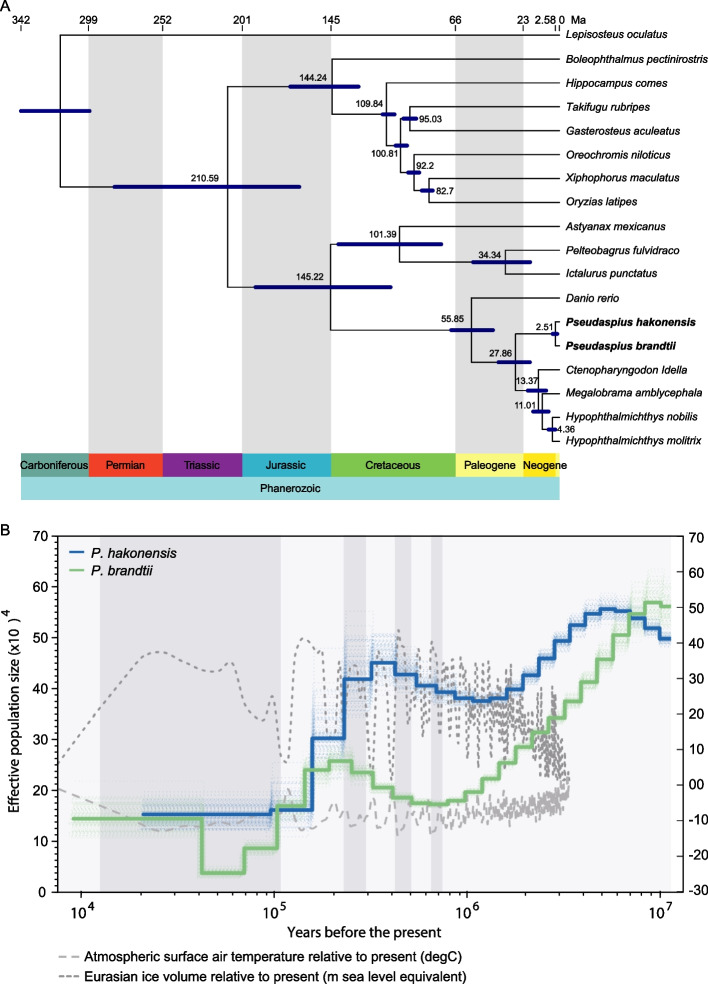
Phylogenetic analysis and demographic histories of *P. hakonensis* and *P. brandtii*. **A** Phylogenetic tree and divergence times estimated for the *P. brandtii*, *P. hakonensis*, and other 16 teleosts. Error bars indicate 95% confidence levels. *P. hakonensis* and *P. brandtii* were highlighted in bold. **B** Demographic histories were reconstructed using PSMC. Blue lines represent *P. hakonensis*, and green lines represent *P. brandtii*. Dark grey box represents glacial periods, and French grey box represents interglacial periods

We conducted a phylogenetic analysis based on 1597 single-copy orthologous genes to explore the evolutionary relationships and divergence times of *P. hakonensis*, *P. brandtii*, and 16 other teleost fishes (Additional file 2: Figure S2). The time-calibrated phylogeny showed that *P. hakonensis* and *P. brandtii* formed a sister clade to grass carp (*Ctenopharyngodon idella*), bighead carp (*Hypophthalmichthys nobilis*), silver carp (*H. molitrix*), and blunt snout bream (*Megalobrama amblycephala*). The two clades shared a most recent common ancestor (MRCA) approximately 27.86 million years ago (Ma) during the Palaeogene period (Fig. 2A). The divergence time between *P. hakonensis* and *P. brandtii* was estimated to be 2.51 million years ago (Ma). These results shed light on the evolutionary history and genetic relationships of these two Far Eastern daces and their place within the broader teleost fish lineage.

The population history was reconstructed by calculating the heterozygosity of the genomes of two species using pairwise sequentially Markovian coalescent (PSMC) model [34]. The population demography analysis of the two species exhibited a similar starting point around 10^4^ to 10^7^ years ago, but thereafter, they followed divergent trends. These patterns could be correlated with significant climatic oscillations, including glacial-interglacial cycles and sea level changes. Such environmental factors might have played a crucial role in shaping the population dynamics and distribution of these Far Eastern daces over time (Fig. 2B). We observed that the first population contraction occurred around 6 million years ago (Ma), followed by a remarkable and massive expansion around 0.9 Ma, reaching its peak at approximately 0.6 Ma during the largest Quaternary glaciation (0.8–0.2 Ma). Subsequently, a second contraction phase began. These fluctuations in population size over geological timeframes shed light on the species’ responses to major climatic events and offer valuable insights into its evolutionary history and adaptive strategies. Similarly, the size of the *P. brandtii* population decreased around 9 Ma, followed by a population expansion at approximately 0.7 Ma and peaked around 0.4 Ma during the largest Quaternary glaciation (0.8–0.2 Ma). Our analysis suggests that both *Pseudaspius* species populations experienced multiple bottlenecks that correlated with glacial activities. Specifically, the population declines coincided with the onset of warm interglacial periods when deglaciation dramatically reduced the available habitat. Notably, throughout various periods of evolution, the effective population size of *P. hakonensis* remained relatively higher and more stable compared to *P. brandtii*. This observation may be attributed to *P. hakonensis*’ robust adaptability and resistance to changing environments. These population dynamics and differences in effective population size provide valuable insights into the responses and resilience of the two *Pseudaspius* species to historical climatic changes and the complex interplay between environmental conditions and their evolutionary strategies.

### Gene family expansion and contraction

The expansion and contraction of gene families can have a significant impact on speciation and environmental adaptation [35]. The analysis of gene family evolution showed that 148 gene families were expanded, while 837 gene families were contracted in the common ancestor of *P. hakonensis* and *P. brandtii*, respectively (Additional file 2: Figure S3). Based on the results, further enrichment analysis was performed using the GO and KEGG databases. The expanded gene families were significantly enriched in 174 GO terms and 16 KEGG pathways (Additional file 2: Figure S4A and S4B), such as protein-DNA complex subunit organization (GO:0071824), nucleosome (GO:0000786), DNA packaging (GO:0006323), chromatin assembly (GO:0031497), DNA conformation change (GO:0071103), natural killer cell mediated cytotoxicity (ko04650), glycosylphosphatidylinositol (GPI)-anchored proteins (ko00537), and CD molecules (ko04090) that associated with cell growth and metabolism and cell damage repair.

Conversely, the contraction gene families were significantly enriched in 645 GO terms and 61 KEGG pathways (Additional file 2: Figure S4C and S4D), such as negative regulation of proteolysis (GO:0045861), activation of immune response (GO:0002253), cell adhesion molecules (ko04514), and phagosome (ko04145) that associated with immune response. These results suggested that these biological processes of gene family expansion and contraction in the two *Pseudaspius* species may be associated with their adaptation to the seawater.

### Adaptation to osmoregulation

**Fig. 3:**
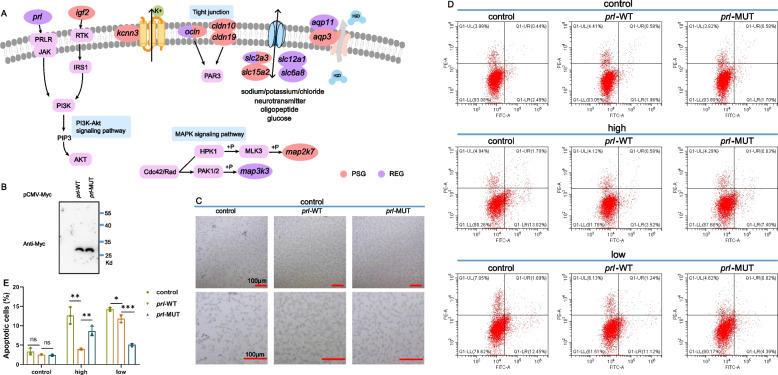
Genomic evidence of osmoregulation related genes in *P. hakonensis* and *P. brandtii*. **A** Genes and pathways involved in osmoregulation are shown in the colored circles and light blue box, respectively. PSGs are marked in pink circles, REGs are marked in purple circles. **B** Immunoblotting assay of *prl* in HEK293T cells. HEK293T cells were cultured in 6-well plates and transfected with indicated plasmids (3 μg each) for 24 h, and then the cells were harvested to perform immunoblotting. **C** *prl*-WT reduces cell crumbling and abscission after high osmotic pressure treatment. HEK293T cells were cultured in 6-well plates and transfected with indicated plasmids (3 μg each) for 8 h and then cultured under high osmolarity condition (600 mOsmol/kg) for 16 h and imaged under a microscope Nikon TE2000-U. **D**–**E** *prl*-WT reduces cell apoptosis in high osmolarity condition (600 mOsmol/kg) or low osmolarity condition (200 mOsmol/kg) detected by flow cytometry analysis (**D**), and statistical analyses were performed using the GraphPad Prism software (7.0) (**E**). HEK293T cells were cultured in 6-well plates and transfected with the indicated plasmids (3 μg each) for 8 h and then cultured under high osmolarity condition (600 mOsmol/kg) for 16 h (high group) or cultured under high osmolarity condition (600 mOsmol/kg) for 12 h followed by low osmolarity condition (200 mOsmol/kg) for 4 h (low group). Data show mean ± SD; Student’s two-tailed *t* test. *P < 0.05, **P < 0.01, ***P < 0.001, data from three independent experiments

Migratory fish exhibit the capacity to persist in both freshwater and high salinity seawater environments by employing osmotic regulation mechanisms. These regulatory processes are essential for maintaining cellular homeostasis and achieving internal environmental balance [36]. The branch-site model was employed to identify the genes evolving under positive selection for seawater adaptation. Among the 7194 single-copy orthologous genes across seven species, a total of 355 positively selected genes (PSGs) were identified in the ancestor of *P. hakonensis* and *P. brandtii* genomes (Additional file 2: Figure S5). We found nine PSGs were strongly associated with osmoregulation, including *cldn10*, *igf2*, *slc2a3*, *kcnn3*, *ocln*, *map2k7*, *aqp3*, *slc15a2*, and *cldn19* (Fig. 3A, Table 1, Additional file 1: Table S9), presumably reflecting the seawater adaptation of *P. hakonensis* and *P. brandtii*. Additionally, we detected 704 rapidly evolving genes (REGs) in the ancestor of *P. hakonensis* and *P. brandtii* genomes. Among them, seven genes related to osmoregulation were found, including *ocln*, *slc2a3*, *prl*, *aqp11*, *slc6a8*, *slc12a1*, and *map3k3* (Fig. 3A, Table 2, Additional file 1: Table S9).

**Table 1:** Positively selected genes with osmoregulation of both *P. hakonensis* and *P. brandtii*

Gene id in *P. hakonensis*	Gene id in *P. brandtii*	Gene name	Description	*P*-value	Adjusted *P*-value
evm.model.Contig196.29	evm.model.Contig8.162	*ocln*	Occludin	0.002161284	0.027519075
evm.model.Contig117.16	evm.model.Contig11.383	*igf2*	Insulin-like growth factor 2	0.000854549	0.016889081
evm.model.Contig27.200	evm.model.Contig55.130	*slc2a3*	Solute carrier family 2, facilitated glucose transporter member 3	0.000650832	0.013650395
evm.model.Contig56.10	evm.model.Contig252.6	*cldn10*	Claudin-10	0.000285494	0.006418262
evm.model.Contig101.4	evm.model.Contig3.11	*aqp3*	Aquaporin-3	0	0
evm.model.Contig1.321	evm.model.Contig4.99	*slc15a2*	Solute carrier family 15 member 2-like	3.99E − 07	1.45E − 05

**Table 2:** Rapid evolution genes with osmoregulation and opsin of both *P. hakonensis* and *P. brandtii*

Gene id in *P. hakonensis*	Gene id in *P. brandtii*	Gene name	Description	*P*-value	Adjusted *P*-value
evm.model.Contig196.29	evm.model.Contig8.162	*ocln*	Occludin	0.002161284	0.027519075
evm.model.Contig27.200	evm.model.Contig55.130	*slc2a3*	Solute carrier family 2, facilitated glucose transporter member 3	4.60E − 06	0.000387153
evm.model.Contig5.58	evm.model.Contig13a.236	*prl*	Prolactin	1.10E − 05	0.000741856
evm.model.Contig154.2	evm.model.Contig6c.48	*aqp11*	Aquaporin-11-like	0.000135301	0.004177491
evm.model.Contig2.156	evm.model.Contig45.24	*rh1*	Rhodopsin	0.000710964	0.013148265

Among these important genes, *ocln* and *slc2a3* genes had both rapid evolution and positive selection (Fig. 3A, Table 1 and Table 2). The gene *slc2a3*, namely the facilitative Na^+^-independent sugar transporters, which contributes to osmoregulatory activities in the fish kidney [37], showed positive selection and accelerated evolution. It was found with A42E and V147L specific amino acid site mutations in both *P. hakonensis* and *P. brandtii*, these mutations occurred in the MFS_1 domain and were likely to have an impact on the three-dimensional structure of the protein (Additional file 2: Figure S6A and S6B). Another gene, *cldn10*, which participates in the creation and maintenance for cation-selective pore junctions in salt-secreting tissues of teleost fishes [38], was found with Y79H and L92T specific amino acid site mutations which occurred in the Claudin_2 domain in both *P. hakonensis* and *P. brandtii*, and this was also likely to affect the three-dimensional structure (Additional file 2: Figure S6A and S6C).

The *prl* gene directly contributes to the survival and functionalization of branchial ion cells involved in osmoregulation (Additional file 2: Figure S6D) [39]. To further investigate whether rapid evolution of *prl* (*prolactin*) in *Pseudaspius* is involved in their salinity adaptation, we first cloned and tested the expression of *prl* wild-type (*prl*-WT) and mutant (*prl*-MUT) (Additional file 2: Figure S6D). Immunoblotting showed that both *prl*-WT and *prl*-MUT could be detected in transfected HEK293 cells (Fig. 3B). Given that hypertonicity induced by high bay salt, the main component of which is NaCl, decreases cell volume, increases cytosolic osmolality, and alters mitochondrial osmotic equilibrium (Michea et al., 2002), we analyzed whether *prl* gene prevents the corresponding hypertonic threat. As expected, cells treated with high osmolarity (600 mOsmol/kg) showed significant shrinkage and shedding, and then overexpression of *prl*, especially overexpression of *prl*-WT, significantly improved the cell status (Fig. 3C). Previous studies have shown that changes in osmolarity induce cells to enter the apoptotic program (Michea et al., 2002; Dmitrieva et al., 2000). To further elucidate the effect of *prl* on osmolarity-induced apoptosis of HEK293 cells, flow cytometry analysis was performed. As shown in Fig. 3D, E, apoptosis was significantly induced by high osmolarity (600 mOsmol/kg), in contrast to overexpression of *prl*-MUT, overexpression of *prl*-WT significantly inhibited osmolarity-induced cell apoptosis. Taken together, the results suggest that the *Pseudaspius prl* gene enhanced cell adaptation to altered osmolarity.

### Vision adaptation

In addition to osmotic regulation, freshwater and marine transition also require a suite of adaptations in other physiological and life history traits, including diet, metabolism, defense from predators, reproductive strategies, and even vision (Van Nynatten et al., 2021). The spectra of available light differ significantly between marine and freshwater habitats. Light that penetrates the open ocean is known to shift towards the blue end of the spectrum with increasing depth (Jerlov, 1968). In contrast, the light spectrum in riverine environments attenuates more rapidly, is more spectrally diverse, but has been less explored concerning the visual systems of riverine animals (Levine and MacNichol, 1979; Costa et al., 2013). These distinctions in the aquatic light environment are believed to impact the visual systems of fishes, leading to freshwater fishes generally exhibiting more redshifted visual pigments (Rennison et al., 2016; Musilova et al., 2019).

**Fig. 4:**
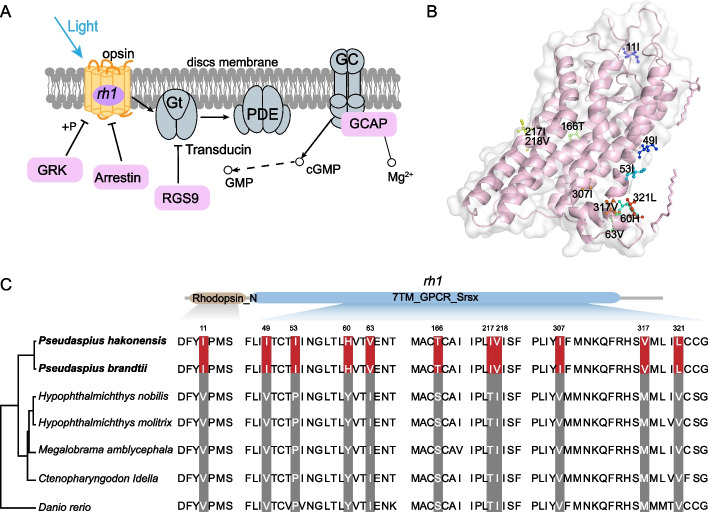
Genomic evidence of visual opsin genes in *P. hakonensis* and *P. brandtii*. **A** Genes involved in phototransduction cascade are shown in the colored circles; a REG was marked in purple circle. **B** The three-dimensional structure of RH1 protein. **C** The *rh1* gene had specific AA replacement in *P. hakonensis* and *P. brandtii* based on sequence alignments

Rhodopsin (*rh1*), the light-sensitive visual pigment expressed in rod photoreceptors, is specialized for vision in dim-light environments and has been confirmed to be associated with marine to freshwater croaker invasion (Van Nynatten et al., 2021) and was also reported to play an important role in migratory eels [40, 41]. Our results showed that the *rh1* gene involved in phototransduction cascade exhibited a rapid evolutionary rate that may have implications in adapting to distinct light environments (Fig. 4A, Table 2). Subsequently, *rh1* gene underwent 11 specific amino acid replacements which occurred in the Rhodopsin_N and 7TM_GPCR_Srsx domains in both *P. hakonensis* and *P. brandtii*, and these amino acid mutation sites also located in the key position of the functional domain (Fig. 4B, C). Therefore, the accelerated evolution of the *rh1* gene is likely to be closely associated with the diverse light conditions encountered during the migration of *P. hakonensis* and *P. brandtii*.

### Conserved non-coding elements (CNEs) linked to osmoregulation

**Fig. 5:**
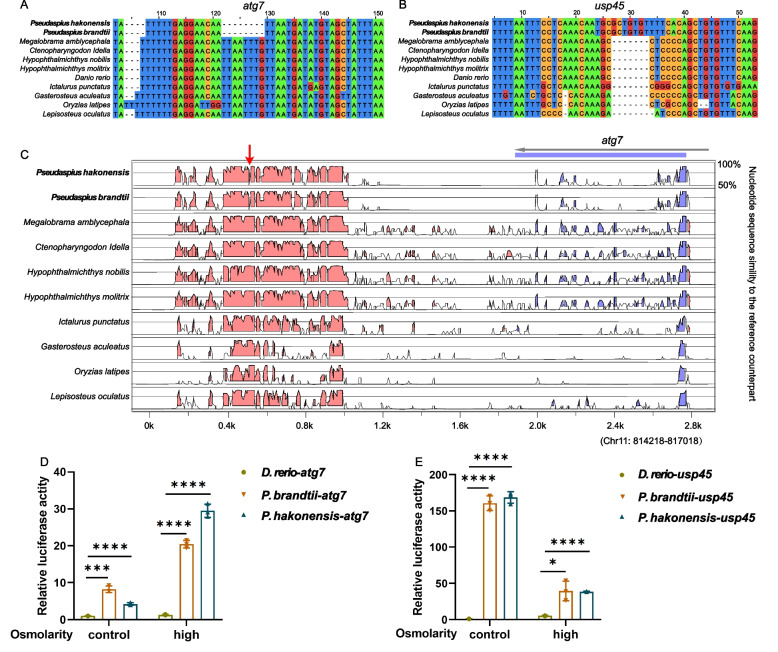
CNE sequence analysis that may be related to osmoregulation. **A** An 8-bp specific deletion of *P. hakonensis* and *P. brandtii* was located near target gene *atg7*. **B** A 7-bp specific insertion of *P. hakonensis* and *P. brandtii* was located near target gene *usp45*. **C** VISTA sequence conservation plot of the *P. hakonensis* and *P. brandtii* specific deletion CNE around *atg7*, using zebrafish as reference. Red arrows indicate missing bp. **D**, **E** Luciferase activity assays of the fragments of *atg7* (**D**) and *usp45* (**E**) promoter from *Danio rerio*, *P. brandtii*, and *P. hakonensis* under control (315 mOsmol/kg) or high osmolarity conditions (600 mOsmol/kg) in HEK293 cells. *P < 0.05, **P < 0.01, ***P < 0.001, ****P < 0.0001

CNEs are usually distributed in the upstream and downstream regions of genes and served as the core components of gene regulatory networks to regulate gene expression [42]. To determine the extent of CNEs loss or gain in *P. hakonensis* and *P. brandtii*, we predicted genome wide CNEs with reference to the zebrafish genome. Consequently, we successfully identified 18,581 CNEs across 11 teleosts through syntenic alignments. We further analyzed the CNEs that were specifically deleted or inserted in *P. hakonensis* and *P. brandtii* and found that they were present in the neighborhood of 175 genes with 196 CNE sequences, including *atg7* and *usp45* genes related to osmoregulation. In particular, there was one CNE (~ 160 bp) near *atg7* with an 8-bp specific deletion in the *P. hakonensis* and *P. brandtii* (Fig. 5A, C) and another CNE (~ 180 bp) near *usp45* with a 7-bp specific insertion in the *P. hakonensis* and *P. brandtii* (Fig. 5B) using zebrafish annotations as reference, which may be associated with adaptation to seawater.

To further explore whether the *Pseudaspius-*specific CNEs near *atg7* and *usp45* genes were involved in osmoregulation, we inserted these CNEs into the pGL3-Basic vector and co-transfected them into HEK293T cells, which were cultured under control (315 mOsmol/kg) or high osmolarity conditions (600 mOsmol/kg). Then, their promoter activity was determined using the dual-luciferase reporter assay system. The results of our reporter assay suggest that the upstream fragment of *atg7* and *usp45* from *P. brandtii* and *P. hakonensis* exhibit higher promoter activity compared to corresponding fragments from zebrafish (Fig. 5D, E). Notably, the promoter activity of *P. brandtii* and *P. hakonensis atg7* was significantly induced at high osmolarity (Fig. 5D). Overall, these results demonstrated that the *Pseudaspius-*specific CNEs near *atg7* and *usp45* genes played a significant role in osmoregulation.

## Discussion

Two of the few migratory species cyprinids, *P. hakonensis* and *P. brandtii*, are ideal fish species to investigate the genetic basis of adaptation to seawater [21, 43]. With advancements in sequencing technology, comparative genomics studies leverage available data to reveal the evolutionary patterns of adaptive traits in fishes in special environmental conditions [44–46]. Here, we generated chromosomal level genome assemblies of *P. hakonensis* and *P. brandtii* with 840.77 Mb and 848.49 Mb, respectively. Both of *P. hakonensis* and *P. brandtii* were anchored with 25 chromosomes and had good collinearity with zebrafish. Analysis of population history showed that two species formed sister groups with *C. idella*, *H. nobilis*, *H. molitrix*, and *M. amblycephala* in Cyprinidae. The genomic information of two species will contribute to a deeper understanding of the genetic mechanism of seawater adaptation.

Surviving in diverse aquatic environments requires ability to regulate their osmotic pressure balance, since salinity plays a crucial role in the ecological environment [47]. Previous studies investigated the biological process genes including metabolism, immune system, cell activity, and growth potentially associated with Nile tilapia adaptation to different salinities as well as phenotypic differentiation of morphology, physiology, and behavior between the migratory population and the freshwater resident population of *Coilia nasus* in the process of adaptive evolution [48, 49]. We hypothesized that these biological processes of gene family expansion and contraction in the two *Pseudaspius* species might be linked to the physiological and behavioral changes observed in the two *Pseudaspius* species as they adapted to the evolution of seawater.

In previous studies, hatching enzymes associated with the evolution of salinity adaptation has been identified in a whole genome of *Oryzias javanicus,* revealing the genetic mechanism of adaptation to different salinities [32]. Similarly, the Atlantic sturgeon has the physiological capability to move between salinity habitats with varying salinity. Tests on its growth and osmotic regulation have indicated its adaptability to these changing salinity conditions. [50]. In the current study, our analyses yielded a list of adaptively evolving genes, which comprised 355 PSGs and 704 REGs in the ancestor node of *P. hakonensis* and *P. brandtii*. Notably, several genes related to osmotic regulation were identified as PSGs and REGs. The candidate genes *ocln*, *cldn10* and *cldn19* encoded the components of a tight connection (Fig. 3A). Previous studies have provided evidence identifying the *ocln* gene as an osmoregulation-related gene in *Acipenser baerii* [51], while the *cldn19* gene played significant roles in osmoregulatory physiology of *Petromyzon marinus* [52]. Moreover, the PSG *cldn10*, which predominantly expressed in osmoregulatory organs of teleost fishes [38, 53], was found have two specific AA replacements in the two *Pseudaspius* species (Additional file 2: Figure S6A). In addition, osmotic regulation affected the expression of many SLCs (solute carriers), such as *slc15a2*, *slc6a8*, *slc12a1*, and *slc2a3* [24, 54–56]. For instance, *slc2a3* gene was important in the fish kidney for osmoregulatory activities [56]. Here, *slc15a2*, *slc6a8*, *slc12a1*, and *slc2a3* genes were involved in material (such as sodium/potassium/chloride, neurotransmitter, oligopeptide, and glucose) exchange (Fig. 3A). Importantly, *slc2a3* is identified as a both positively selected and rapidly evolving gene that had two specific amino acid replacements in the MFS_1 domain (Additional file 2: Figure S6A and 6B). Meanwhile, several ion transporters and genes were also important in osmoregulation, such as *kcnn3* that has been found to be upregulated in an euryhaline pupfish adaptation to high salinity conditions [57] (Fig. 3A).

Aquaporins (AQPs) are crucial for rapid osmoregulation by facilitating the diffusion of water and osmolytes across cellular membranes [58]. Recent studies suggested that the *aqp3* gene potentially contributes to water metabolism and osmotic pressure regulation in *Lateolabrax maculatus* [59]. Moreover, hypertonic conditions have resulted in increased expression of *aqp11* in *Clarias magur* hepatocytes [60, 61]. In particular, our genomic analyses showed that *aqp3* and *aqp11* genes may modulate the exchange of water molecules between the inside and outside of the cell membrane (Fig. 3A). We also found *igf2* to be under selection, which influences osmoregulation by stimulating Na + /K + -ATPase activity and promoting chloride cell differentiation (Chandhini et al., 2021) (Fig. 3A). A *prl* gene has been showed to have a reduced expression level following seawater adaptation in *Oncorhynchus rhodurus* (Yada et al., 2010) (Fig. 3A). The *prl* and *igf2* genes bind to receptors and transmit signals to PI3K, participating in the PI3K-Akt signaling pathway (Fig. 3A). It has been demonstrated that Mitogen-activated protein kinase (MAPK) cascades are major participants in osmotic stress response of fish, and are involved in regulating cell proliferation, growth, death, and other physiological processes [62–64]. The expression of the *map2k7* gene has been showed to be upregulated in the muscle of *Penaeus monodon* during acute hyposaline stress, and *map3k3* also contributes to the activation of the osmoprotective transcription factor *nfat5* in response to high NaCl [65, 66]. The genes, in particular *map2k7* and *map3k3*, part of MAPK signaling pathway, were identified as PSG and REG in the two *Pseudaspius* species, respectively (Fig. 3A). The role of PRL in osmoregulation has been discovered as early as 1950s by researching on killifish (*Fundulus heteroclitus*), with *F. heteroclitus* unable to survive in freshwater following hypophysectomy while PRL treatment allowed hypophysectomized killifish to survive [67]. Subsequently, several studies found a direct contribution of PRL to osmoregulation by knocking out this gene in zebrafish (Shu et al., 2016). However, it is still unclear whether the rapid evolving of *prl* gene in two *Pseudaspius* species contribute to their adaptation to seawater. In our study, by integrating comparative genomics and functional assays, we found that the rapid evolving of *prl* gene is indeed associated with their osmoregulation in the two *Pseudaspius* species. Osmoregulatory candidate genes are potentially important that contribute to the adaptation of *Pseudaspius* species to seawater.

Previous studies demonstrated that visual systems have undergone diversification in migratory fish [68]; freshwater eels changed their spectral sensitivity by switching chromophore types or expressing different opsin genes in response to changing light environments during migration from fresh water to deep sea environments [68, 69]. Moreover, cloning and expression analysis of freshwater and deep-sea opsin genes in the eel *A. japonica* have revealed that the rod-shaped visual pigment (*rh1*) changes from “freshwater” form to “deep-sea” form during maturation [70, 71]. The *rh1* underwent positive selection during salmonid evolution, linked to divergent light environments [72]. Interestingly, our genomic analyses found that *rh1* is a rapidly evolving gene with several amino acid replacements (Fig. 4). Therefore, the genetic innovations may be associated with the seawater light environment of *P. hakonensis* and *P. brandtii*.

Many CNEs function as cis-regulatory elements, such as enhancers, repressors, and insulators, and the presence or absence of CNEs plays a significant role in phenotypic differences and morphological innovation [73–75]. Notably, our genomic analyses found that there were CNEs loss near the *atg7* and obtain close to *usp45* (Fig. 5). Thus, we propose CNEs play pivotal roles in osmoregulation during seawater adaptation of *P. hakonensis* and *P. brandtii*. Previous reports have indicated adverse effects of hypotonic stress on various developmental stages of *Macrobrachium rosenbergii* larvae; the expression of the autophagy-related gene *atg7* showed significant changes under hypotonic stress [76]. In salinity-induced transcriptome studies of marine and freshwater threespine stickleback, a decrease in *usp45* expression was observed 6 h after the sudden exposure to seawater [77]. Notably, our genomic analyses found that there were CNEs loss near the *atg7* and obtain close to *usp45* (Fig. 5). Thus, we propose that this may play pivotal roles in osmoregulation and will yield insights into seawater adaptation of *P. hakonensis* and *P. brandtii*.

## Conclusions

We acquired high-quality chromosome-level genome assemblies of *P. hakonensis* and *P. brandtii*. Genome evolution analysis provided insights into genetic changes related to osmoregulation and vision that may be critical to the seawater adaptation in *P. hakonensis* and *P. brandtii*. Our results provide opportunities to elucidate the molecular mechanisms underlying the evolutionary adaptations of migratory fish to seawater and present a valuable resource for future research on adaptive evolution in *Pseudaspius* species.

## Methods

### Sample DNA and RNA extraction, sequencing

*P. hakonensis* individual and *P. brandtii* individual were collected from Suifen River in Dongning City, Heilongjiang Province, China. Genomic DNA was extracted from the muscle using Puregene Tissue Core 81 Kit A (Qiagen, Maryland, USA). DNA integrity was checked using 1% agarose gel electrophoresis and Pultton DNA/Protein Analyzer (Plextech, Berkeley, USA). Total RNA was extracted from tissues of *P. hakonensis* and *P. brandtii*, including muscle, eye, heart, liver, kidney, gill, spleen, spermary, swim bladder, and brain, by using TRIzol reagent (Invitrogen, USA). RNA quality was checked with a NanoDrop ND-1000 spectrophotometer (Labtech, Palaiseau, France) and a 2100 Bioanalyzer (Agilent Technologies, Waldbronn, Germany).

A paired-end library with inset sizes of 150 bp was constructed according to the Illumina protocol and sequenced on the NovaSeq 6000 platform (Illumina) to assess the complexity of the genome, correction of the genome assembly, and assembly evaluation. Hi-C libraries were constructed and sequenced to obtain a chromosomal-level genome assembly. After reads with low-quality bases (reads with more than 10% N bases or low-quality bases ≤ 5), adapter sequences, and duplicated sequences were discarded, the clean reads were used for subsequent analysis. Nanopore libraries were generated according to the manufacturer’s instructions and sequenced on 13 flow cells using the GridION X5 DNA sequencer (Oxford Nanopore). For the Nanopore reads, the mean quality of each read was calculated, and any reads with mean quality > 7 were retained for subsequent analyses. An SMRTbell library with a fragment size of 20 kb was constructed using a SMRTBell template preparation kit 1.0 (PacBio) by following the manufacturer’s protocol, and the library was sequenced with the PacBio Sequel II system. Then, full-length refined consensus transcripts were generated using the PacBio Iso-Seq pipeline (https://github.com/PacifcBiosciences/IsoSeq).

### Genome size estimation and genome assembly

All the filtered Illumina pair-end reads were used for k-mer frequency analysis [78]. The genomic size was estimated on the basis of the following formula: *G* = k-mer number/k-mer depth, where *G* represents the genome size, where k-mer number are the total number, and k-mer depth represents the average depth of 17-mers, respectively. After the Nanopore low-quality reads were filtered, the remaining high-quality reads were first calibrated using the CANU (version 1.5) [79] and then assembled using the genome assembler FLYE (version 2.6) [80] with default parameters. The original Nanopore reads were polished for three rounds using racon (version 1.2.1) [81] and further corrected for three rounds using Illumina reads with pilon (version 1.21) [82] for a consistent assembly. The filtered Hi-C reads were first aligned to the assembled genome using the BOWTIE (version 2.2.5) [83]; only the read pairs with both ends uniquely aligned to the genome were selected. Then, the corrected contigs and valid Hi-C reads were used to perform the chromosomal-level genome assembly by LACHESIS [84] with default parameters. In addition, a heatmap based on the interaction signals was constructed to evaluate the quality of the chromosomal-level genome assembly. Finally, the completeness of our final genome assembly was evaluated using BUSCO (version 3) [85].

### Genome annotation

In order to predict the repeat elements in *P. hakonensis* and *P. brandtii*, REPEATMODELER (version 1.0.5) [86] was used to construct a de novo transposable element library, which was then used to predict repeats with REPEATMASKER (version 4.0.6) [86]. In the *P. hakonensis* and *P. brandtii* genomes, ab initio, homologues, and RNA-sequencing methods were used for predicting the protein-coding genes. The above three gene sets were integrated to yield a comprehensive and nonredundant gene set using EVIDENCEMODELER (EVM, version 1.1.1) [87]. Then, the integrated gene set was translated into amino acid sequences and performed gene functional annotations by searching against known databases, including NR, InterPro, Uniport, Pfam, and eggNOG, with a cutoff *E* value of 1e − 5.

### Phylogenetic analysis

Protein sequences from a total of 18 species including *P. hakonensis* and *P. brandtii* (assembled in this study), *C*. *Idella* [88], *H. nobilis*, *H. molitrix* [89], *M. amblycephala* [90], *D. rerio*, *Astyanax mexicanus* [91], *Ictalurus punctatus* [92], *Pelteobagrus fulvidraco* [93], *Gasterosteus aculeatus* [94], *Takifugu rubripes* [95], *Oreochromis niloticus* [96], *O. latipes* [97], *Xiphophorus maculatus* [98], *Hippocampus comes* [99], *Boleophthalmus pectinirostris* [100], and *Lepisosteus oculatus* [101] were constructed by ORTHOMCL (version 2.0.9) with default settings, and 1597 single-copy orthologues were identified. Then, the protein sequences of single-copy orthologous genes were further aligned using MUSCLE [102] with default parameter settings. The maximum likelihood method was used to construct a phylogenetic tree using RAXML (version 8.1.19) [103] with 1000 bootstraps under the GTR + I model. MCMCTREE of the PAML package [104] was used to calculate the divergence times among *P. hakonensis* and *P. brandtii* and 16 other teleost fishes, and the fossil records were obtained from TIMETREE database (http://www.timetree.org/) for calibration; we set four calibration time points (*C. Idella*–*L. oculatus*: ~ 298.8–342.5 MYA; *C. Idella*–*D. rerio*: ~ 41.7–68.9 MYA; *H. comes*–*G. aculeatus*: ~ 99.5–116.7 MYA; *O. niloticus*–*X. maculatus*: ~ 81.2–91.6 MYA).

### Demographic history

We concluded the demographic history for the *P. hakonensis* and *P. brandtii* by pairwise sequentially Markovian coalescent (PSMC) analysis [34]. Heterozygote sites across the genome were used to estimate changes in effective population size. To generate consensus diploid sequences for the two individuals separately by SAMtools (version 1.3.1) [105], the “fq2psmcfa” tool was used to create the input file for PSMC modeling, with the option -q20. Moreover, the effective population history was inferred using PSMC with 100 bootstraps and plotted using the psmc_plot.pl pipeline. The generation time for two species was set as 4 years, and the mutation rate was 1.3387e − 09 per year per nucleotide for *P. hakonensis* and 1.2125e − 09 per year per nucleotide for *P. brandtii*. In addition, we obtained the atmospheric surface air temperature (°C) and Eurasian ice volume (m sea level equivalent) data for the past 3 million years from the NCEI (http://www.ncdc.noaa.gov/).

### Gene family expansion and contraction

To determine gene family expansion and contraction in the *P. hakonensis* and *P. brandtii* genomes, CAFÉ (version 3.1) [106] was used with default parameter settings. If the copy number of the *P. hakonensis* and *P. brandtii* were higher or lower than those found in their close ancestral clades, then we identified the gene family as a substantially expanded or contracted gene family. A conditional *P*-value for each gene family was calculated, and gene modules of the significantly expanded and contracted gene family were extracted and were subjected to GO and KEGG functional enrichment analyses with a false discovery rate (FDR) adjusted *P*-value < 0.05, respectively. During functional enrichment, the zebrafish genes were selected as background. GO terms or KEGG pathways with a *P*-value < 0.05 were considered significantly enriched.

### Identification of positively selected genes and rapidly evolving genes

Single-copy orthologous genes were used in the detection of PSGs and REGs. The branch-site model incorporated in the PAML (version 4.8) package [107] was employed to detect PSGs in *P. hakonensis* and *P. brandtii* genomes, with the non-migrated fishes as the background. The null model used in the branch-site test assumed that the comparison of the substitution rates at nonsynonymous and synonymous sites (Ka/Ks ratio) for all codons in all branches must be ≤ 1, whereas the alternative model assumed that the foreground branch included codons evolving at Ka/Ks > 1. The *P*-values were computed based on the chi-square statistic adjusted by the false discovery rate (FDR) method, and genes with adjusted *P*-value < 0.05 were considered as PSGs. We identified REGs in the *P. hakonensis* and *P. brandtii* genomes using the branch model in the CODEML program in PAML. The likelihood ratio test (LRT) was employed to discriminate between alternative models for each orthologue with df (degree of freedom) = 1. We treated the orthologue as evolving with a significantly faster rate in foreground branch if the FDR adjusted *P*-value < 0. 05 and a higher ω (Ka/Ks) ratio in the foreground branch than in the background branches.

### Identification of specific amino acid mutations and protein structure simulation

The homologous protein sequences of *P. hakonensis* and *P. brandtii* and other fishes were aligned and compared. A particular amino acid mutation was defined as the *P. hakonensis* and *P. brandtii* amino acid were different from acids of all other species including *C. Idella* [88], *H. nobilis*, *H. molitrix* [89], *M. amblycephala* [90], and *D. rerio*. Missing sites may be present in the protein sequences of species with poorly assembled genomes because of assembly quality among different genomes. Therefore, the specific amino acid mutation was considered only if at least 80% of species had valid amino acid information. Then, it was determined whether the mutated amino acids located in the protein domain of the *P. hakonensis* and *P. brandtii* using Pfam [108].

First, we preliminarily predicted the *slc2a3*, *cldn10*, and *rh1* protein structures based on the amino acid sequence on the SWISS-MODEL online website and downloaded the PDB file (https://swissmodel.expasy.org/). Next, the predicted result with the maximal score was selected as the best structure and visualized by Pymol (http://www.PyMOL.org/).

### Identification of conserved noncoding elements

To identify CNEs, using the *D. rerio* genome as a reference, whole genome alignments for 11 teleosts were generated, and the genome data were downloaded from ENSEMBL; they were *P. hakonensis* and *P. brandtii* (assembled in this study), *C*. *Idella*, *H. nobilis*, *H. molitrix*, *M. amblycephala*, *D. rerio*, *I. punctatus*, *G. aculeatus*, *O. latipes*, and *L. oculatus*. First, the results of the LAST whole genome alignment were submitted to the LAST subroutine “Maf-swap” [109], and the sequence in the maf format alignment was changed; then, the MULTIZ v3 subroutine “roast” [110] was used to extract sequence alignments at the same location on the chromosomes of different species. Next, further analyses were run with subprograms of PHAST [111]. “msa_view” was used to identify aligned 4D sites of alignments with parameters “–informat MAF –4d –features.” We estimated a neutral phylogenetic model using “phyloFit” [112]. We estimated rho (expected substitution rate of conserved elements relative to neutrality) using “phastCons” with “–estimate-rho –no-postprobs–most-conserved –score.” Conserved models for each window were combined by phyloBoot [112] and then used for initial conserved element prediction. The coding regions and non-coding regions of each gene based on the GFF3 file of the *D. rerio* genome were separated by using ANNOVAR [113] after conserved elements had been identified. Finally, a series of python scripts were used to detect the divergent CNEs and divergent coding genes. A final set of 18,581 CNEs with a minimum of 30-bp in length were identified. We mainly focused on CNEs which are located in the intergenic regions, in introns, and within the 10-kb upstream or downstream flanking regions of genes. Finally, the VISTA [114] was used to plot the alignments after determining the required CNE sequence.

### Detection of apoptotic cells

HEK293T cells were cultured under different osmolarity conditions as indicated. HEK293T cells were cultured in 6-well plates and transfected with indicated plasmids (3 μg each) for 8 h and then cultured under high osmolarity condition (600 mOsmol/kg) for 16 h and imaged under a microscope Nikon TE2000-U. For flow cytometry analysis, cells were harvested and stained with FITC Annexin V and PI using the FITC Annexin V Apoptosis Detection Kit I (556,547, BD Pharmingen) according to the manufacturer’s instructions. The detection of apoptotic cells was performed using the Beckman CytoFLEXS, and the data were analyzed using the CytExpert software.

### Dual-luciferase assay

The chemically synthesized fragments of specific CNEs near *Danio rerio atg7* (NC: 007122.7_814119-815,755), *P. brandti atg7* (Chromosome 21: 1,036,539–1038266) and *P. hakonensis atg7* (Chromosome 21: 42,224–43,950), *D. rerio usp45* (NC: 007127.7_32717090-32718855), *P. brandti usp45* (Chromosome 10: 21,314,205–21315977), and *P. hakonensis usp45* (Chromosome 10: 20,933,460–20935232) were inserted into the pGL3-Basic vector (Promega, USA) and were sequenced (Sangon Biotech, China). The HEK293T cells were seeded in 24-well plates for 24 h and then co-transfected with 200 ng constructed pGL3-Basic vectors and 3 ng pRL-CMV as an internal control using VigoFect (Vigorous Biotech). Sixteen hours after transfection, cells were cultured under control (315 mOsmol/kg) or high osmolarity conditions (600 mOsmol/kg) for 8 h and then harvested using passive lysis buffer. Luciferase activity in cell extracts was determined using a dual-luciferase reporter assay system (Promega) according to the manufacturer’s protocol. The light output of the transcription activity was divided by the output of the Renilla luciferase activity for sample normalization.

### Statistical analysis

All statistical analyses were performed using the GraphPad Prism software (7.0). Results with error bars represent mean ± SD. Statistical analysis was performed using Student’s two-tailed *t*-test. A *P*-value less than 0.05 was considered significant. Statistical significance is indicated as follows: *P < 0.05, **P < 0.01, ***P < 0.001, ****P < 0.0001.

### Supplementary Information

**Additional file 1: Table S1.** The statistics of sequencing of *P. hakonensis* and *P. brandtii* genome. **Table S2.** The statistics of the assembly of *P. hakonensis* and *P. brandtii* genome. **Table S3.** Comparison of the genome assembly quality from fishes in Leuciscidae. **Table S4.** Statistics of the pseudochromosome assemblies using Hi-C data. **Table S5.** BUSCO analysis result of *P. hakonensis* and *P. brandtii* genome. **Table S6.** Repeat elements in the *P. hakonensis* and *P. brandtii* genome with Repeatmasker. **Table S7.** Genetic structure characteristics of the *P. hakonensis* and *P. brandtii* compared to other fish species. **Table S8.** Gene function annotation of *P. hakonensis* and *P. brandtii*. **Table S9.** Positively selected genes and rapid evolution genes with osmoregulation of both *P. hakonensis* and *P. brandtii*.

**Additional file 2: Figure S1.** Genome-wide Hi-C heatmap of *P. hakonensis* (A) and *P. brandtii* (B). The blocks represent the 25 pseudochromosomes. The color bar illuminates the contact density from high (red) to low (white) in the plot. **Figure S2.** Comparison of gene family clusters. The horizontal axis display species and the vertical axis display genes number. **Figure S3.** Expanded/contracted gene families for *P. hakonensis* and *P. brandtii* and another 16 teleosts with the divergence time. **Figure S4.** GO and KEGG enrichment analysis of expanded/contracted gene families for *P. hakonensis* and *P. brandtii*. (A) Top 20 go_term of expanded gene families for *P. hakonensis* and *P. brandtii*. (B) Top 16 KEGG pathway of expanded gene families for *P. hakonensis* and *P. brandtii*. (C) Top 20 go_term of contracted gene families for *P. hakonensis* and *P. brandtii*. (D) Top 20 KEGG pathway of contracted gene families for *P. hakonensis* and *P. brandtii*. **Figure S5.** Phylogenetic tree for positive selective genes analysis. **Figure S6.** Genomic evidence of osmoregulation related genes in *P. hakonensis* and *P. brandtii*. (A) The *slc2a3* and *cldn10* genes had specific AA replacement in *P. hakonensis* and *P. brandtii* based on sequence alignments. (B) The three-dimensional structure of SLC2A3 protein. (C) The three-dimensional structure of CLDN10 protein. (D) Alignment of PRL amino acid sequences showing specific amino acid mutations in the *Pseudaspius prl* gene.

**Additional file 3. ** Uncropped blots.

## Data Availability

Raw sequencing reads have been deposited in NCBI with the BioProject accession PRJNA980574 [115]. Uncropped blots are available in Additional file 3.

## Abbreviations

CNEs: Conserved non-coding elements
Gb: Gigabase
Mb: Megabase
BUSCO: Benchmarking Universal Single-Copy Orthologs
NR: Non-Redundant Protein Sequence Database
MRCA: Most recent common ancestor
Ma: Million years ago
PSMC: Pairwise sequentially Markovian coalescent
GO: Gene Ontology
KEGG: Kyoto Encyclopedia of Genes and Genomes
PSGs: Positively selected genes
REGs: Rapidly evolving genes
WT: Wild-type
AQPs: Aquaporins
