# Supplementary material for: Genomic insights into the seawater adaptation in Cyprinidae
Source: BMC Biol. 2024 Apr 19;22:87. doi: 10.1186/s12915-024-01885-2 (PMC11027309; doi:10.1186/s12915-024-01885-2)
Supplement: Supplementary file 1 — Additional file 1: Table S1. The statistics of sequencing of P. hakonensis and P. brandtii genome. Table S2. The statistics of the assembly of P. hakonensis and P. brandtii genome. Table S3. Comparison of the genome assembly quality from fishes in Leuciscidae. Table S4. Statistics of the pseudochromosome assemblies using Hi-C data. Table S5. BUSCO analysis result of P. hakonensis and P. brandtii genome. Table S6. Repeat elements in the P. hakonensis and P. brandtii genome with Repeatmasker. Table S7. Genetic structure characteristics of the P. hakonensis and P. brandtii compared to other fish species. Table S8. Gene function annotation of P. hakonensis and P. brandtii. Table S9. Positively selected genes and rapid evolution genes with osmoregulation of both P. hakonensis and P. brandtii. [file 12915_2024_1885_MOESM1_ESM.docx]

**Table S1.** The statistics of sequencing of *P. hakonensis* and *P. brandtii* genome.

| **Sequencing type** | *P. hakonensis* | *P. brandtii* | Total |
| --- | --- | --- | --- |
| Nanopore sequencing | 80.50 Gb | 81.42 Gb | 161.92 Gb |
| Illumina sequencing | 65.78Gb | 68.17Gb | 133.95 Gb |
| Hi-C sequencing | 90.63Gb | 81.85Gb | 172.48 Gb |
| Total | 236.91 Gb | 231.44 Gb | - |

|  | *P. hakonensis* | *P. brandtii* |
| --- | --- | --- |
| GC content | 39% | 38% |
| Minimum length | 11,225 | 15,961 |
| Maximum length | 49,749,698 | 48,486,748 |
| Median length | 36,040 | 214,096 |
| Contig N50 | 3,683,115 | 3,825,663 |
| Total length | 840,772,322 | 848,489,045 |

**Table S2.** The statistics of the assembly of *P. hakonensis* and *P. brandtii* genome.

**Table S3.** Comparison of the genome assembly quality from fishes in Leuciscidae.

| Species | Total length | Contig N50 | Publish date |
| --- | --- | --- | --- |
| *P. hakonensis* | 840,772,322 | 3,683,115 | This study |
| *P. brandtii* | 848,489,045 | 3,825,663 | This study |
| *Rhinichthys klamathensis goyatoka* | 1,065,491,053 | 2,916,613 | 2023/04/27 |
| *Phoxinus dragarum* | 968,145,596 | 128,188 | 2023/11/06 |
| *Tiaroga cobitis* | 1,320,521,697 | 8,259,576 | 2023/08/01 |
| *Squalius squalus* | 1,136,474,712 | 2,697 | 2023/06/07 |
| *Parachondrostoma miegii* | 972,309,943 | 2,090 | 2023/04/26 |
| *Vimba vimba* | 1,054,310,786 | 7,031,647 | 2022/04/06 |
| *Semotilus atromaculatus* | 1,099,257,308 | 30,568,897 | 2023/09/21 |
| *Pseudochondrostoma polylepis* | 907,194,594 | 1,691 | 2023/04/26 |

**Table S4.** Statistics of the pseudochromosome assemblies using Hi-C data.

| Pseudo-chromosomes | *P. hakonensis*  Chr length (bp) | *P. brandtii*  Chr length (bp) |
| --- | --- | --- |
| chr1 | 49,747,198 | 48,484,348 |
| chr2 | 46,711,325 | 48,191,485 |
| chr3 | 42,520,034 | 43,249,746 |
| chr4 | 38,136,637 | 39,328,125 |
| chr5 | 37,600,928 | 37,275,392 |
| chr6 | 35,788,564 | 35,615,369 |
| chr7 | 33,865,985 | 35,503,898 |
| chr8 | 33,823,628 | 34,998,144 |
| chr9 | 32,615,688 | 33,605,092 |
| chr10 | 32,327,439 | 32,453,601 |
| chr11 | 32,073,663 | 32,241,519 |
| chr12 | 31,970,695 | 32,103,563 |
| chr13 | 31,476,126 | 31,531,747 |
| chr14 | 31,441,486 | 31,530,906 |
| chr15 | 31,418,276 | 31,307,738 |
| chr16 | 30,209,725 | 31,163,431 |
| chr17 | 29,686,101 | 30,796,302 |
| chr18 | 29,632,660 | 29,444,905 |
| chr19 | 28,689,604 | 29,388,704 |
| chr20 | 28,491,676 | 28,884,010 |
| chr21 | 26,644,089 | 27,588,302 |
| chr22 | 24,883,981 | 25,996,036 |
| chr23 | 24,404,676 | 24,915,599 |
| chr24 | 23,805,491 | 24,822,559 |
| chr25 | 23,648,041 | 24,274,344 |

**Table S5.** BUSCO analysis result of *P. hakonensis* and *P. brandtii* genome.

|  | *P. hakonensis* | | *P. brandtii* | |
| --- | --- | --- | --- | --- |
| Term | Gene number | Percentage | Gene number | Percentage |
| Complete BUSCOs | 4,334 | 94.50% | 4,349 | 94.90% |
| Complete and single-copy BUSCOs | 4,036 | 88.00% | 4,030 | 87.90% |
| Complete and duplicated BUSCOs | 298 | 6.50% | 319 | 7.00% |
| Fragmented BUSCOs | 133 | 2.90% | 108 | 2.40% |
| Missing BUSCOs | 117 | 2.60% | 127 | 2.70% |
| Total BUSCO groups searched | 4,584 | 100% | 4,584 | 100% |

**Table S6.** Repeat elements in the *P. hakonensis* and *P. brandtii* genome with Repeatmasker.

| Type | *P. hakonensis* | | *P. brandtii* | |
| --- | --- | --- | --- | --- |
|  | Length (bp) | % in genome | Length (bp) | % in genome |
| DNA | 169286102 | 20.13 | 167655966 | 19.76 |
| LINE | 24919450 | 2.96 | 23945520 | 2.82 |
| SINE | 7568151 | 0.90 | 5028218 | 0.59 |
| LTR | 32878418 | 3.91 | 38117583 | 4.49 |
| Unclassified | 75834627 | 9.02 | 80879869 | 9.53 |
| Total | 310486748 | 36.93 | 315627156 | 37.20 |

**Table S7.** Genetic structure characteristics of the *P. hakonensis* and *P. brandtii* compared to other fish species.

| Species | Number of gene | Average gene length (bp) | Average CDS length (bp) | Average exon length (bp) | Average intron length(bp) |
| --- | --- | --- | --- | --- | --- |
| *Takifugu rubripes* | 21,411 | 11,809.09 | 1,892.94 | 193.40 | 2,315.37 |
| *Oryzias latipes* | 22,127 | 17,999.42 | 1,784.94 | 280.37 | 3,134.68 |
| *Lepisosteus oculatus* | 18,341 | 25,099.34 | 1,792.12 | 245.43 | 4,321.86 |
| *Danio rerio* | 25,281 | 31,135.21 | 1,543.62 | 242.93 | 5,820.02 |
| *Tribolodon hakonensis* | 23,829 | 18,760.55 | 1,784.38 | 168.08 | 1,753.32 |
| *Tribolodon brandtii* | 24,012 | 18,804.12 | 1,784.33 | 168.56 | 1,933.00 |

**Table S8.** Gene function annotation of *P. hakonensis* and *P. brandtii*.

| **Annotation database** | **Annotated number of predicted genes** | |
| --- | --- | --- |
|  | ***P. hakonensis*** | ***P. brandtii*** |
| NR | 22,980 | 23,190 |
| Uniprot | 20,251 | 20,344 |
| Pfam | 21,679 | 21,903 |
| eggNOG | 22,462 | 22,633 |
| Interpro | 20,977 | 21,191 |
| All annotated | 23,252 | 23,458 |

| **Gene id in *P.* *hakonensis*** | **Gene id in** ***P. brandtii*** | **Gene name** | **Description** | ***P*-value** | **Adjusted *p*-value** |
| --- | --- | --- | --- | --- | --- |
| evm.model.Contig103.41 | evm.model.Contig17.137 | *kcnn3* | small conductance calcium-activated potassium channel protein 2-like isoform X2 | 0 | 0 |
| evm.model.Contig155.8 | evm.model.Contig138.35 | *map2k7* | dual specificity mitogen-activated protein kinase kinase 7-like isoform X2 | 3.87E-06 | 0.000122235 |
| evm.model.Contig145.14 | evm.model.Contig76.54 | *cldn19* | claudin-19-like isoform X3 | 2.42E-06 | 8.06E-05 |
| evm.model.Contig86.101 | evm.model.Contig65.92 | *slc6a8* | sodium- and chloride-dependent creatine transporter 1-like isoform X1 | 0.000407565 | 0.009077469 |
| evm.model.Contig355.2 | evm.model.Contig428.6 | *slc12a1* | solute carrier family 12 member 1 | 0.001824057 | 0.0245276 |
| evm.model.Contig4a.43 | evm.model.Contig19.159 | *map3k3* | mitogen-activated kinase kinase kinase 3-like protein | 1.41E-05 | 0.000883173 |

**Table S9.** Positively selected genes and rapid evolution genes with osmoregulation of both *P. hakonensis* and *P. brandtii*.
