# Supplementary material for: Genomic insights into the seawater adaptation in Cyprinidae
Source: BMC Biol. 2024 Apr 19;22:87. doi: 10.1186/s12915-024-01885-2 (PMC11027309; doi:10.1186/s12915-024-01885-2)
Supplement: Supplementary file 2 — Additional file 2: Figure S1. Genome-wide Hi-C heatmap of P. hakonensis (A) and P. brandtii (B). The blocks represent the 25 pseudochromosomes. The color bar illuminates the contact density from high (red) to low (white) in the plot. Figure S2. Comparison of gene family clusters. The horizontal axis display species and the vertical axis display genes number. Figure S3. Expanded/contracted gene families for P. hakonensis and P. brandtii and another 16 teleosts with the divergence time. Figure S4. GO and KEGG enrichment analysis of expanded/contracted gene families for P. hakonensis and P. brandtii. (A) Top 20 go_term of expanded gene families for P. hakonensis and P. brandtii. (B) Top 16 KEGG pathway of expanded gene families for P. hakonensis and P. brandtii. (C) Top 20 go_term of contracted gene families for P. hakonensis and P. brandtii. (D) Top 20 KEGG pathway of contracted gene families for P. hakonensis and P. brandtii. Figure S5. Phylogenetic tree for positive selective genes analysis. Figure S6. Genomic evidence of osmoregulation related genes in P. hakonensis and P. brandtii. (A) The slc2a3 and cldn10 genes had specific AA replacement in P. hakonensis and P. brandtii based on sequence alignments. (B) The three-dimensional structure of SLC2A3 protein. (C) The three-dimensional structure of CLDN10 protein. (D) Alignment of PRL amino acid sequences showing specific amino acid mutations in the Pseudaspius prl gene. [file 12915_2024_1885_MOESM2_ESM.docx]

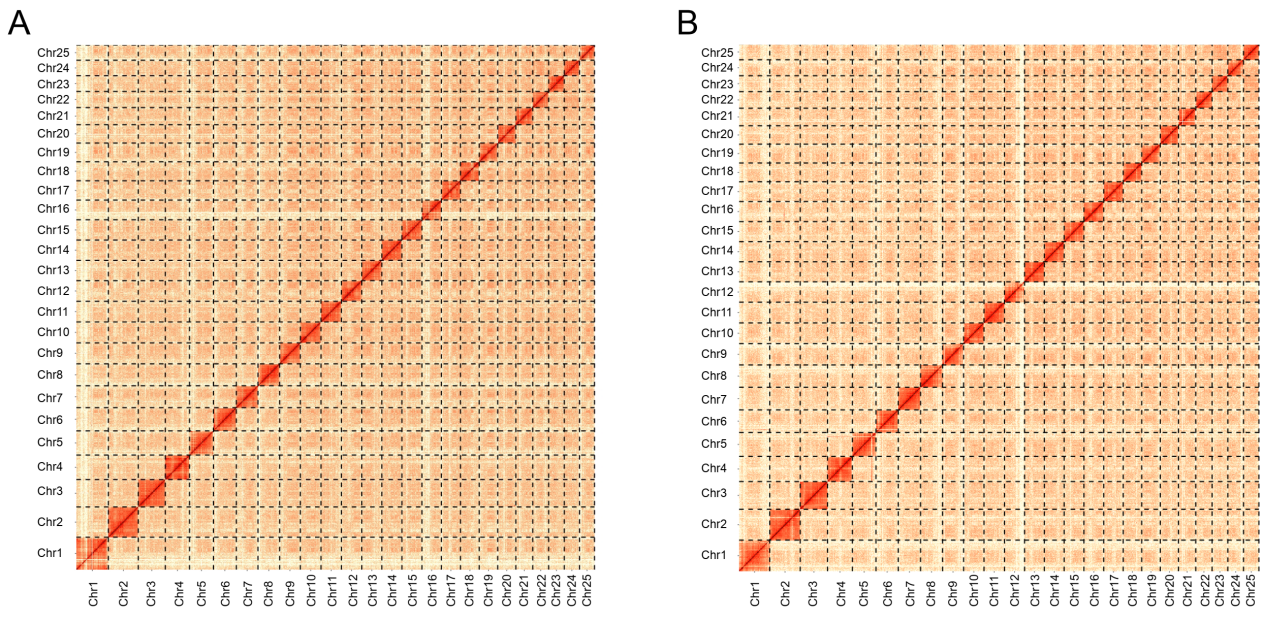


**Figure S1.** Genome-wide Hi-C heatmap of *P. hakonensis* (A) and *P. brandtii* (B). The blocks represent the 25 pseudochromosomes. The color bar illuminates the contact density from high (red) to low (white) in the plot.


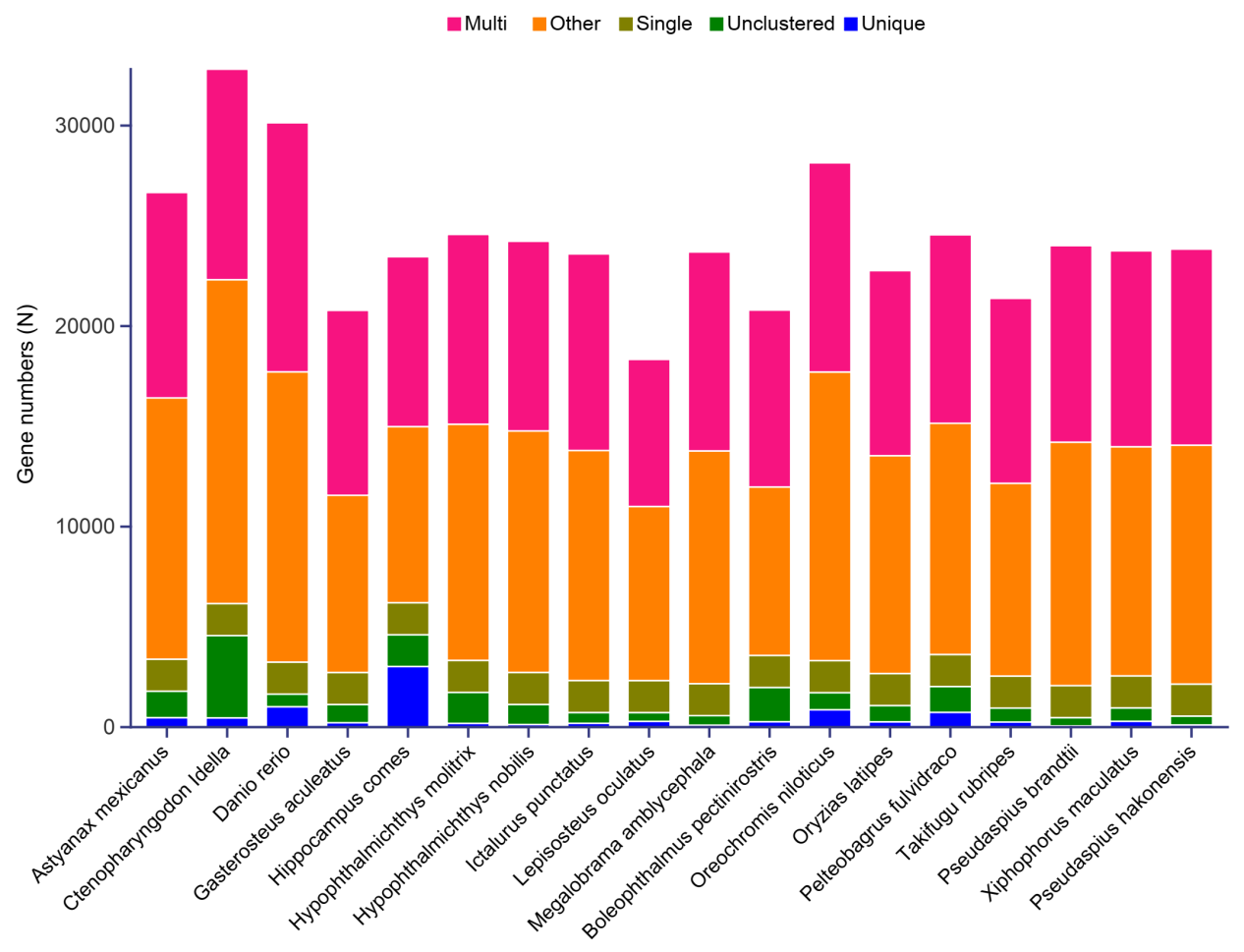


**Figure S2.** Comparison of gene family clusters. The horizontal axis display species and the vertical axis display genes number.


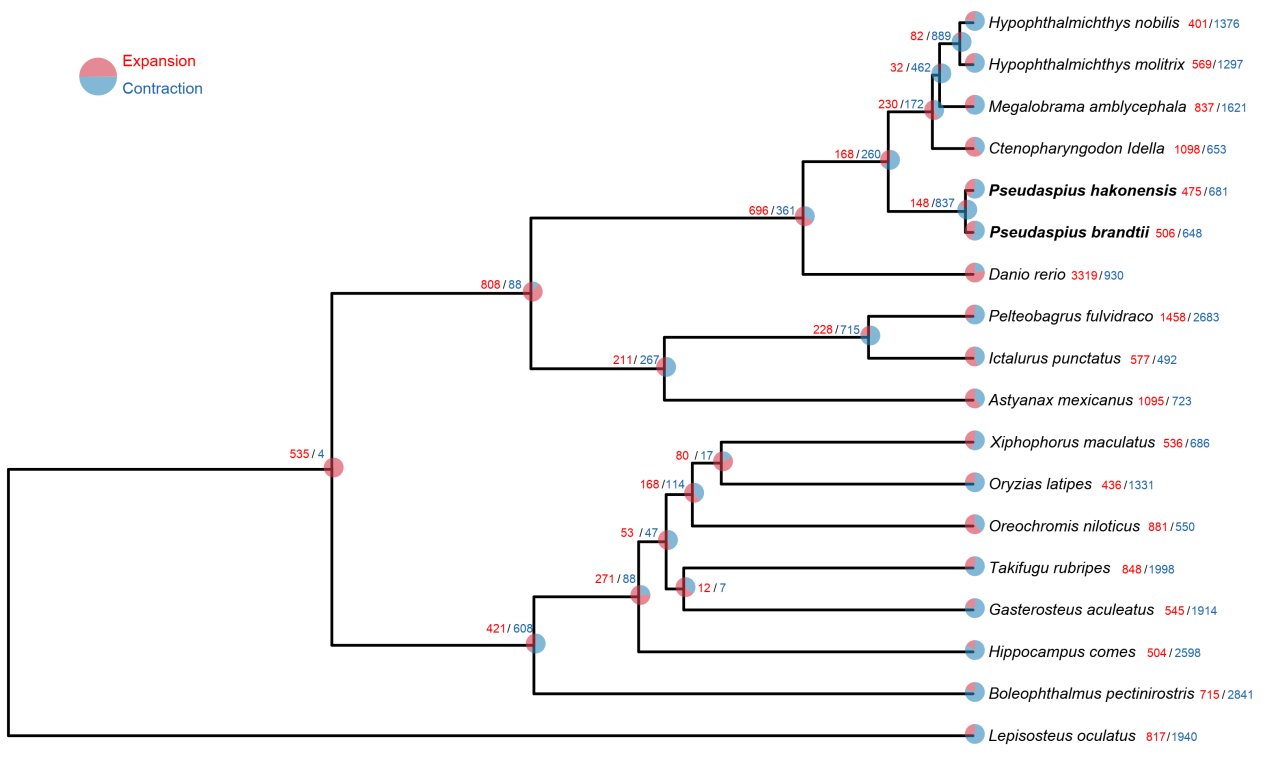


**Figure S3.** Expanded/contracted gene families for *P. hakonensis* and *P. brandtii* and another 16 teleosts with the divergence time.


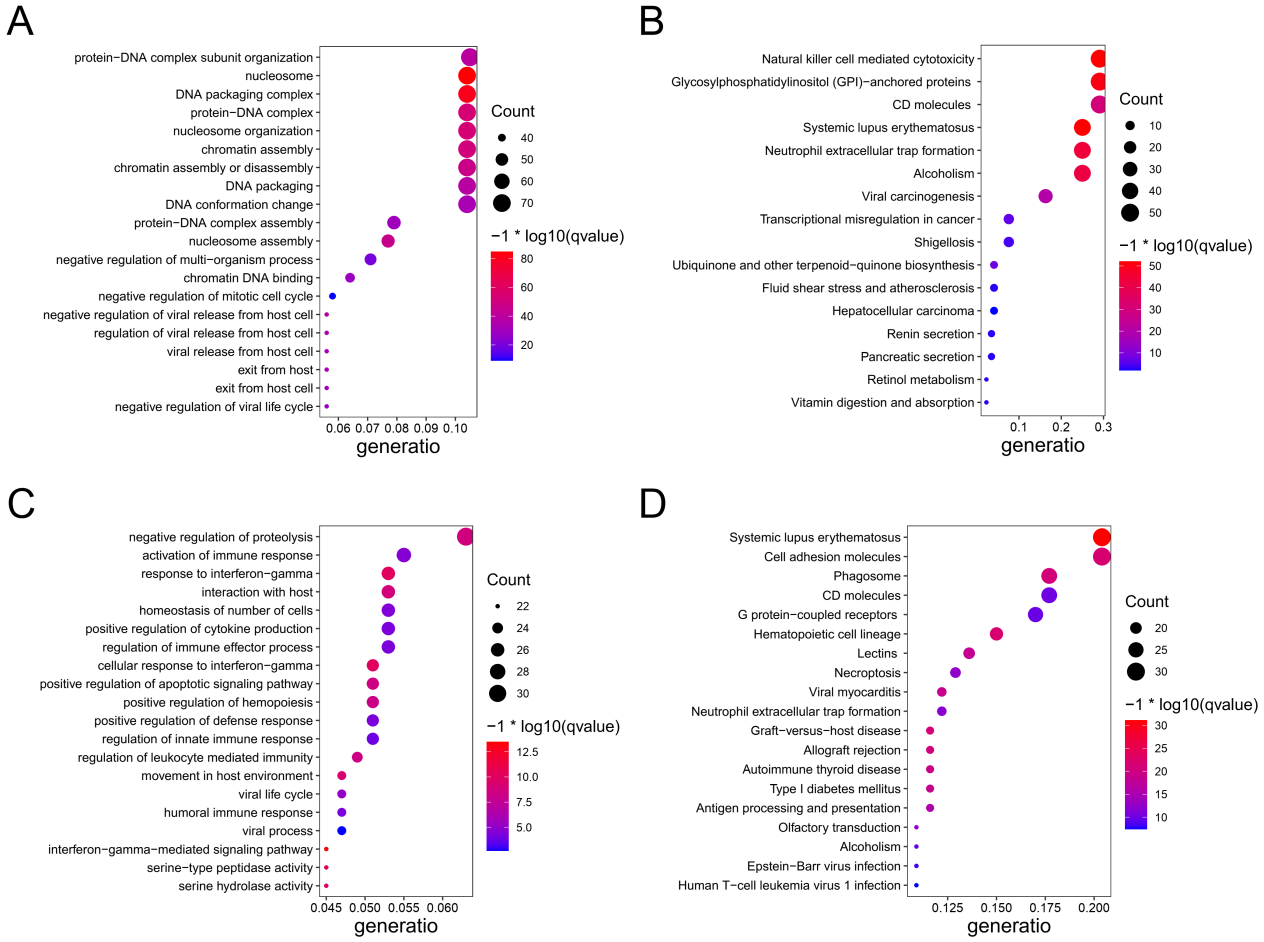


**Figure S4.** GO and KEGG enrichment analysis of expanded/contracted gene families for *P. hakonensis* and *P. brandtii*. (A) Top 20 go_term of expanded gene families for *P. hakonensis* and *P. brandtii*. (B) Top 16 KEGG pathway of expanded gene families for *P. hakonensis* and *P. brandtii*. (C) Top 20 go_term of contracted gene families for *P. hakonensis* and *P. brandtii*. (D) Top 20 KEGG pathway of contracted gene families for *P. hakonensis* and *P. brandtii*.


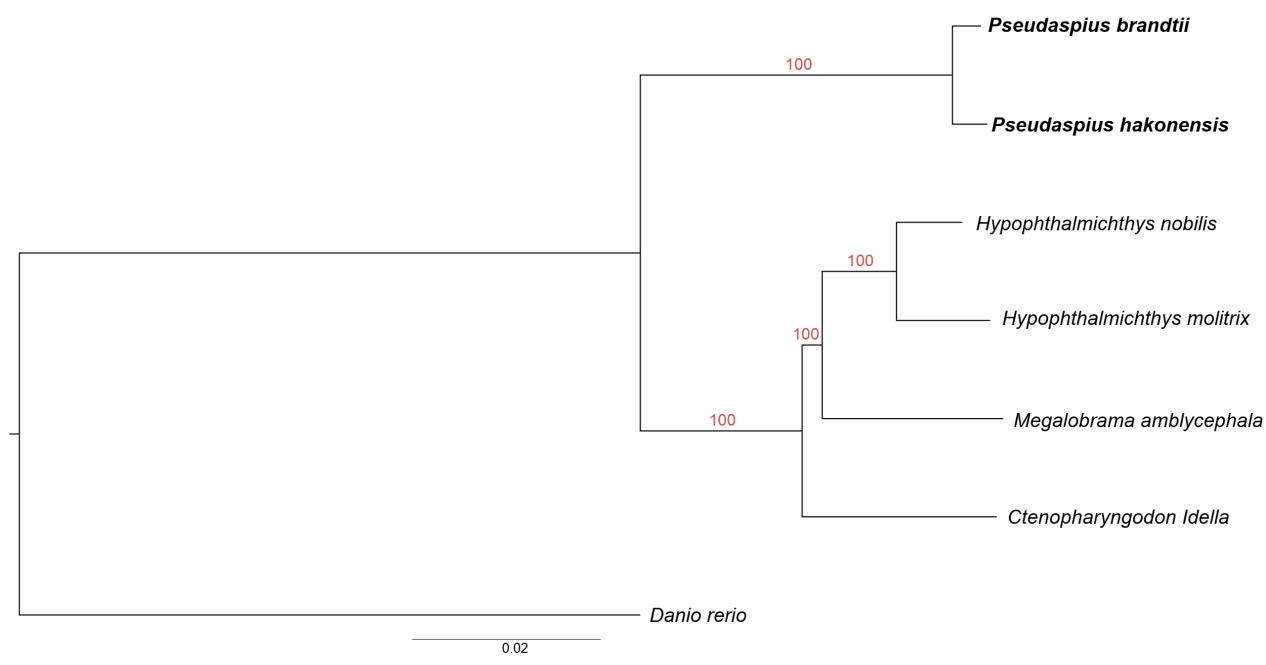


**Figure S5.** Phylogenetic tree for positive selective genes analysis.


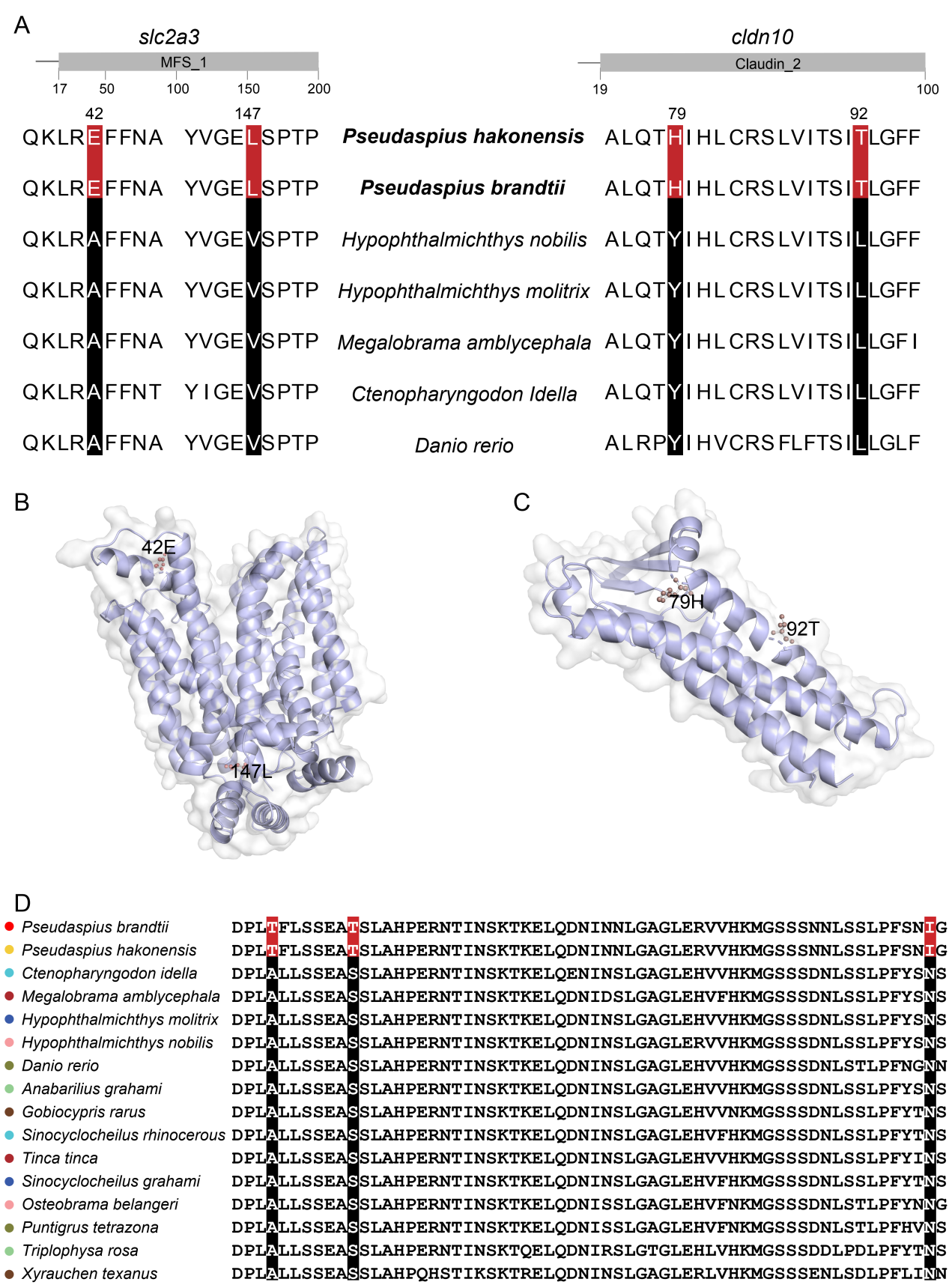


**Figure S6.** Genomic evidence of osmoregulation related genes in *P. hakonensis* and *P. brandtii*. (A) The *slc2a3* and *cldn10* genes had specific AA replacement in *P. hakonensis* and *P. brandtii* based on sequence alignments. (B) The three-dimensional structure of SLC2A3 protein. (C) The three-dimensional structure of CLDN10 protein. (D) Alignment of PRL amino acid sequences showing specific amino acid mutations in the *Pseudaspius prl* gene.
