# Supplementary figures and images for: Genomic insights into the seawater adaptation in Cyprinidae
Source: BMC Biol. 2024 Apr 19;22:87. doi: 10.1186/s12915-024-01885-2 (PMC11027309; doi:10.1186/s12915-024-01885-2)

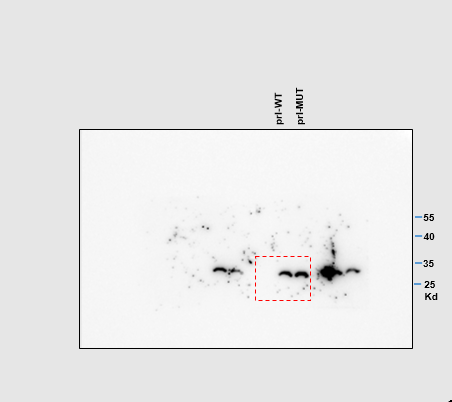

Supplement: Supplementary file 3 — Additional file 3. Uncropped blots. [file 12915_2024_1885_MOESM3_ESM.jpg]
